# Efficacy and Improved Resistance Potential of a Cofactor-Independent InhA Inhibitor of Mycobacterium tuberculosis in the C3HeB/FeJ Mouse Model

**DOI:** 10.1128/AAC.02071-18

**Published:** 2019-03-27

**Authors:** Gregory T. Robertson, Victoria A. Ektnitphong, Michael S. Scherman, Matthew B. McNeil, Devon Dennison, Aaron Korkegian, Anthony J. Smith, Jason Halladay, David S. Carter, Yi Xia, Yasheen Zhou, Wai Choi, Pamela W. Berry, Weimin Mao, Vincent Hernandez, M. R. K. Alley, Tanya Parish, Anne J. Lenaerts

**Affiliations:** a Mycobacteria Research Laboratories, Department of Microbiology, Immunology and Pathology, Colorado State University, Fort Collins, Colorado, USA; b TB Discovery Research, Infectious Disease Research Institute, Seattle, Washington, USA; c Anacor Pharmaceuticals, Palo Alto, California, USA

**Keywords:** C3HeB/FeJ mice, antimicrobial resistance, caseum, drug development, isoniazid, necrotic lesions, tuberculosis

## Abstract

AN12855 is a direct, cofactor-independent inhibitor of InhA in Mycobacterium tuberculosis. In the C3HeB/FeJ mouse model with caseous necrotic lung lesions, AN12855 proved efficacious with a significantly lower resistance frequency than isoniazid. AN12855 drug levels were better retained in necrotic lesions and caseum where the majority of hard to treat, extracellular bacilli reside. Owing to these combined attributes, AN12855 represents a promising alternative to the frontline antituberculosis agent isoniazid.

## Abstract (precis)

AN12855 is a direct, cofactor-independent inhibitor of InhA in Mycobacterium tuberculosis. In the C3HeB/FeJ mouse model with caseous necrotic lung lesions, AN12855 proved efficacious with a significantly lower resistance frequency than isoniazid.

## INTRODUCTION

**FIG 1:**
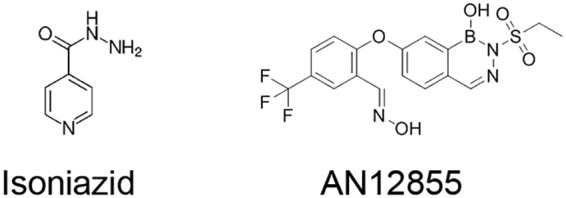
Chemical structures of isoniazid and AN12855.

A previous study described the identification of a novel diazaborine scaffold that inhibits enoyl-ACP reductase (InhA), a clinically proven drug target in Mycobacterium tuberculosis (1). The lead compound ethylsulfonyl benzodiazaborine (AN12855) (Fig. 1) binds to and inhibits InhA with submicromolar affinity through a novel cofactor-independent mechanism (1). Unlike isoniazid (INH) (Fig. 1), which requires prodrug activation by the peroxidase-catalase enzyme KatG (2), AN12855 binds directly to InhA, occupying both the cofactor and substrate-binding sites (1). These features impart two direct benefits to the inhibitor, namely, (i) potent activity against common isoniazid-resistant strains with mutations affecting the nonessential KatG activation enzyme (3) and (ii) improved resistance frequency compared with INH, reflecting a unique binding pocket and essentiality of the InhA target (1). Thus, AN12855 retains potent activity against both drug-susceptible and drug-resistant strains of M. tuberculosis, including those with *katG* and *inhA* coding sequence mutations (1). AN12855 is not cytotoxic against HepG2 or THP-1 cell lines, shows no tolerability issues in mice at doses up to 200 mg/kg of body weight, and exhibits *in vivo* efficacy following oral delivery in murine acute and chronic tuberculosis (TB) efficacy models (1). Given the proven clinical utility of the target, its unique cofactor-independent mode-of-action, and retained activity against clinically relevant *katG* and *inhA* INH-resistant mutants, the diazoborine AN12855 is viewed as a novel compound with utility as both a possible replacement for INH in the standard frontline regimen but also for inclusion in combination with newer regimens under development. Owing to its established mode-of-action, it is anticipated that this will translate into faster killing early, and by having this early killing activity, could lead to treatment shortening when used in combination with other drugs. Thus, AN12855 was reported to represent a promising lead compound for the development of novel TB therapeutics.

**FIG 2:**
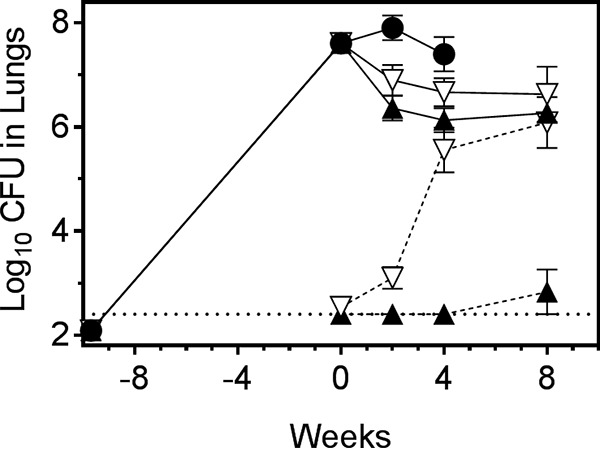
AN12855 at 100 mg/kg is more effective and exhibits lower emergence of drug resistance than isoniazid at 25 mg/kg in C3HeB/FeJ mice with advanced lung pathology. Mice were infected by low-dose aerosol with Mycobacterium tuberculosis Erdman 68 days prior to the start of treatment (week 0). Treatments were administered 5 of 7 days per week, Monday to Friday. AN12855 was formulated in 0.1% (vol/vol) polysorbate-80 and 1% (vol/vol) methyl cellulose 400 cP. Isoniazid was formulated in sterile water. Solid lines depict lung CFU burdens. Dashed lines depict drug-resistant escape mutants. Closed circles, untreated controls. Open triangles, isoniazid treated. Closed triangles, AN12855 treated. The horizontal dotted line represents the lower limit of detection for drug-resistant isolate detection.

A hallmark of active M. tuberculosis infection in humans is the development of pulmonary granulomas with central caseous necrosis (4–8). Unlike conventional TB mouse efficacy models, where granulomas in lungs are exclusively cellular, the C3HeB/FeJ mouse model develops well-defined necrotic lesions with caseous centers that more closely resemble human lesions upon a M. tuberculosis infection (4, 9–11). To begin to understand the impact of advanced lung pathology on treatment outcome, we employed this mouse model to evaluate efficacy, resistance development, and tissue distribution more comprehensively for these distinct InhA inhibitors *in vivo*. In an initial study, we compared the efficacy in lungs of C3HeB/FeJ mice following 2, 4, and 8 weeks of treatment with INH or AN12855. In addition, the emergence of drug resistance was studied during therapy. Thereto, female C3HeB/FeJ mice were infected by low dose aerosol with M. tuberculosis Erdman, as described (12, 13). Lung burdens increased from a mean CFU count of 2.09 (standard error of the mean [SEM], 1.54) log_10_ 1 day following infection to an average of 7.61 (SEM, 0.18) log_10_ at the start of treatment on day 68 (Fig. 2). Gross pathology analysis revealed heterogeneous cellular and caseous necrotic lesions in the lungs of individual mice (11), indicating the desired pulmonary pathology had fully developed prior to the start of treatment (data not shown). Groups of eight mice each were treated as described (1) (Fig. 2). INH given at 25 mg/kg in sterile water reduced lung burdens from the start of treatment by 0.71 logs at 2 weeks and by 0.94 and 0.98 logs at weeks 4 and 8, respectively (Table 1; Fig. 2). These differences were not statistically significant (*P* > 0.05). AN12855 given at 100 mg/kg in 1% (wt/vol) methyl cellulose and 0.1% (vol/vol) polysorbate-80 showed greater bactericidal activity by promoting significant reductions in lung burdens from the start of treatment of 1.25 logs at 2 weeks (*P* < 0.001) and 1.48 (*P* = 0.001) and 1.34 logs (*P* < 0.05) at weeks 4 and 8, respectively (Table 1; Fig. 2). The better overall activity of AN12855 in this model was reproducible in a second independent C3HeB/FeJ study (data not shown). The improved activity of AN12855 over INH in C3HeB/FeJ mice is in contrast to the earlier reported results in the acute gamma interferon knockout (GKO) mouse model or in chronically infected BALB/c mice, where similar efficacy was achieved for INH and AN12855 (1). Thus, in the results presented here, AN12855 showed superior efficacy compared with INH at the prescribed doses in the C3HeB/FeJ mouse model with advanced lung pathology.

**TABLE 1:** Efficacy and drug resistance of AN12855 and isoniazid in the C3HeB/FeJ mouse model

Group	Time point sampled			
	Start of treatment	2 weeks	4 weeks	8 weeks
Lung efficacy [mean ± SEM, *n*]				
Vehicle only	7.61 ± 0.18, 8/8	7.91 ± 0.24, 7/7	7.40 ± 0.33, 7/7	nd^*a*^
Isoniazid (25 mg/kg)		6.90 ± 0.29, 8/8	6.67 ± 0.27, 7/7	6.63 ± 0.53, 7/7
AN12855 (100 mg/kg)		6.36 ± 0.24, 8/8	6.13 ± 0.23, 8/8	6.27 ± 0.11, 8/8
Drug resistance [mean ± SEM, (range), n, % resistance]				
Isoniazid	[≤2.63 ± 0.09, (<2.40–3.00), 6/8, 0.001]	[≤3.10 ± 0.21, (<2.40–3.89), 6/8, 0.016]	[5.56 ± 0.43, (3.24–6.64), 7/7, 8]	[6.09 ± 0.49, (3.77–7.71), 7/7, 29]
AN12855	[<2.40 ± 0.00, (<2.40), 0/8, <0.0006]	[<2.40 ± 0.00, (<2.40), 0/8, <0.011]	[<2.40 ± 0.00, (<2.40), 0/8, <0.019]	[≤2.83 ± 0.43, (<2.40–5.44), 1/8, 0.037]

a nd, not done.

Due to the high bacterial numbers in the C3HeB/FeJ mouse model, it is an ideal model and useful tool for studying resistance frequencies *in vivo*. Earlier published studies have shown a greater propensity for the expansion of drug resistance in C3HeB/FeJ mice compared with M. tuberculosis-infected BALB/c mice (4). We, therefore, considered the hypothesis that the emergence of appreciable drug resistance to INH contributes to the difference in efficacy observed in C3HeB/FeJ mice between both compounds. To test this hypothesis, lung homogenates from each time point were coplated on 7H11-oleic acid-albumin-dextrose-catalase (OADC) agar medium with AN12855 at 1.25 mg/liter or INH at 0.625 mg/liter, representing the lowest selective drug concentrations to give rise to genetically stable drug-resistant isolates using high-titer M. tuberculosis Erdman cultures *in vitro* (1). Additional experimental details are provided in Supplemental Text S1. Consistent with previous studies (4), spontaneous INH-resistance was detected under these conditions at a frequency of 7 × 10^−6^ (i.e., 0.001% INH resistant, as defined above) prior to the initiation of treatment in the lungs of six of eight M. tuberculosis Erdman-infected C3HeB/FeJ mice (Table 1). INH resistance arose at a frequency of 1 × 10^−6^ under the same selection conditions *in vitro*. In contrast, the frequency of AN12855-resistant mutants recovered from M. tuberculosis Erdman-infected C3HeB/FeJ mice prior to the start of treatment was below the limit of detection of <3 × 10^−7^ (<0.0006% AN12855 resistant, as defined above). AN12855 resistance arose at a frequency of 4 × 10^−7^ under identical selection conditions *in vitro*. Similar *in vivo* spontaneous resistance frequencies were observed in a second independent C3HeB/FeJ infection study, indicating the results were reproducible (data not shown).

The initiation of drug treatment with INH in monotherapy resulted in an increase in the proportion of INH-resistant to -susceptible subpopulations such that all of the mice in the INH treatment group had discernible drug-resistant bacilli by the end of treatment (Table 1). The proportion of INH-resistant bacteria within this treatment group increased from 0.001% prior to the initiation of treatment to 29% by the end of treatment (Table 1). As expected, resistance to INH was associated with a spectrum of single nucleotide polymorphisms in the nonessential *katG* gene (i.e., 323A>C, 514G>A [isolated twice], 566A>T, 982T>C, 1431G>A, and 1712G>A) promoting changes in the KatG amino acid sequence (i.e., H108P, A172T [isolated twice], D189V, W328R, W477X, and R571H). This diversity of recovered *katG* alleles, indicates that *in vivo* INH resistance did not arise from one single dominant resistant clone. In contrast, resistance to AN12855 was observed in only one of eight mice, and at the 8-week time point only (Table 1). Although the overall proportion of resistance for AN12855 was much lower than that observed for INH (29% INH resistance compared with 0.037% AN12855 resistance at the end of treatment), the number of AN12855-resistant bacilli observed in this single mouse was high relative to the total bacterial burden (see Table 1). Sequence analysis of five single colony isolates from this mouse revealed a common 287G>T mutation in *inhA*, resulting in InhA_G96V_. Given that a wider spectrum of AN12855-resistant clones have been obtained *in vitro* (i.e., G96A [isolated twice], IG96V, I16T, D148G, P151S, R195Q, I202T, E219A and also a C-15-T mutation in the *fabGI-inhA* promoter [isolated twice]) (1; this study), it seems likely that the resistance observed in this mouse was the result of an expansion of a single AN12855-resistant clone.

The evaluation of an AN12855-resistant clone bearing InhA_G96V_ by broth microdilution MIC (14), revealed a >122-fold shift in MIC to AN12855 but no appreciable shift in MIC for INH (Table 2). In contrast, *in vitro*-isolated M. tuberculosis Erdman bearing a *fabG1-inhA* C-15-T promoter mutation, which increases the cellular abundance of InhA (3, 15), conferred cross-resistance to both INH and AN12855, as expected (Table 2) (1). All MIC values were reproducible in two or more independent studies, and no appreciable MIC shifts were observed for rifampin (≤2-fold). Given the proximity of G96 to the sulfonyl group of AN12855 in the InhA-AN12855 cocrystal (1), we predict that the G96V substitution interferes with docking of AN12855. This is supported by the observation that elevated MICs for AN12855 were also observed for an *in vitro*-isolated AN12855-resistant strain bearing the less bulky InhA_G96A_ substitution (Table 2). Additional studies are required to validate this experimentally. Taken together, these data indicate that AN12855 remains on target *in vivo* and that direct InhA inhibitors as a class of compounds could potentially have reduced potency against the 20% of clinical INH-resistant strains that have mutations in the *fabG1-inhA* promoter (3, 15). These findings should be taken into account when determining doses needed for strain coverage in TB patients.

**TABLE 2:** MICs of M. tuberculosis *inhA* promoter and coding sequence mutants^*a*^

Strain	Source	SNPs		Liquid MIC (μM) of agent:		
		*fabG1-inhA* promoter (nt)	InhA	Rifampin	Isoniazid	AN12855
Erdman	Parent	na	na	0.01	0.44	0.16
ED-DPR18-RM4	*in vivo*	No SNP	G96V	0.007	0.83	>20 (>122)^*b*^
ED-DPR18-RM1	*in vitro*	No SNP	G96A	0.018	0.44	14.2 (87)^*b*^
ED-DPR19-RM2	*in vitro*	C-15T	No SNP	0.02	3.2 (7)^*b*^	1.1 (7)^*b*^

a nt, nucleotide; na, not applicable; SNP, single nucleotide polymorphism; No SNP, no single nucleotide polymorphism.

b Calculated as ∼fold shift versus Erdman.

**FIG 3:**
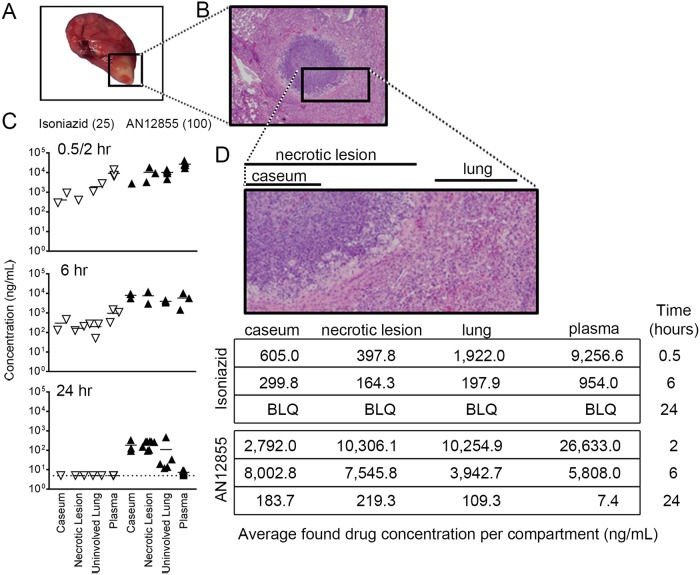
Penetration and retention of isoniazid and the diazaborine AN12855 into different tissue compartments by pharmacokinetic analysis. (A) Gross pathology of a representative Mycobacterium tuberculosis Erdman-infected C3HeB/FeJ lung lobe showing a type I caseous lesion (square). (B) Micrograph of a type I caseous lesion (hematoxylin and eosin stained). (C) Quantitative drug distribution by tissue compartment over time. Mice were sampled after 7 consecutive days of treatment with isoniazid at 25 mg/kg or AN12855 at 100 mg/kg. Time points were 0.5, 6, and 24 h following administration for isoniazid (open triangles) and 2, 6, and 24 h following administration of AN12855 (closed triangles). (D) Mean drug levels by compartment with each drug treatment. The inset shows a magnification of the regions of interest. BLQ, below limits of quantification (<1 ng/ml).

The expansion of diverse INH-resistant subpopulations was, in part, responsible for the more limited efficacy of INH versus the AN12855 group, as roughly 29% of the total bacterial population was INH resistant by the end of treatment. However, it seems unlikely to be the only contributing factor, as differences in efficacy were observed after only 2 weeks of treatment in C3HeB/FeJ mice, which is prior to the significant expansion of INH resistance (see Fig. 2). This prompted us to investigate the systemic and local drug exposure of both drugs by determining their pharmacokinetic (PK) and tissue distribution properties (16) in the C3HeB/FeJ mouse model. For this purpose, mice were divided into treatment groups 10 weeks after aerosol infection, and therapy was administered as above for 7 consecutive days. Plasma, regions of uninvolved lung, whole necrotic type I lesions, and caseum from the core of type I lesions (11) were dissected from individual C3HeB/FeJ mice, as described previously (9). Samples were collected at 0.5 h, 6 h, and 24 h for INH and 2 h, 6 h, and 24 h for AN12855 based on *T*_max_ values from previously published plasma PK studies (1, 17). Results showed that 2 h after dosing, AN12855 drug levels were highest in plasma and uninvolved lung and lowest in necrotic lesions and caseum (Fig. 3C, top panel, filled triangles, and Fig. 3D). Drug levels in all lung compartments were much higher overall for AN12855 than for INH, partly reflecting differences in administered drug doses but also suggesting better drug distribution into tissues/lesions in general (Fig. 3D). As expected, both AN12855 and INH were rapidly cleared from plasma over time (Fig. 3C and D) (1, 17). INH was readily cleared from uninvolved lung, necrotic lesions, and caseum also, with drug levels falling below the limits of quantification by 24 h (Fig. 3D). Contrary to the INH tissue distribution data, AN12855 was found to partition into lung, necrotic lesions, and caseum early and to a remarkably greater extent (Fig. 3C and D). In addition, AN12855 showed selective retention in lung lesions, especially in necrotic lesions and caseum, but not in plasma (Fig. 3C and D), at levels approaching or exceeding the plasma-protein-bound MIC (i.e., 0.5 mg/liter) (1). The data support the hypothesis that the physiochemical properties of AN12855 promote better lesion distribution and retention relative to INH in an animal model presenting with advanced necrotic lung disease. As the majority of extracellular bacilli reside in the caseous necrotic lesion cores of C3HeB/FeJ mice (4), the improved drug exposure inside pulmonary lesions is likely to contribute to the increased efficacy of AN12855 over INH in this TB mouse efficacy model. Other explanations, such as reduced activation of the INH prodrug in hypoxic, necrotic lesions (4, 9–11), seem less likely given that the active INH-NAD+ adduct is readily detected in the Wayne’s anaerobic dormancy model *in vitro* (18).

Our data also suggest a possible advantage of the diazoborine AN12855 with regard to lower resistance development *in vivo*. Although encouraging, placing these resistance data into clinical context will require an evaluation of wild-type MIC distributions to determine a clinically meaningful susceptibility breakpoint for AN12855 in future studies. We note also that the *in vivo* emergence of INH resistance observed herein in C3HeB/FeJ mice more closely resembles that observed in *in vitro* time-kill assays or in the hollow-fiber model (1, 19). These data are in contrast to a complete lack of INH-resistance emergence in M. tuberculosis-infected guinea pigs (20). As INH-resistant isolates are readily detected in sputum of TB infected patients, these findings suggest that the C3HeB/FeJ TB mouse efficacy model might better reflect the human situation in terms of resistance frequency and, therefore, could be a good tool for studying the resistance of single drugs and, potentially, drug combinations for which other models can rarely provide data. Collectively, these combined data support a model that better drug distribution/retention of the diazaborine AN12855 in necrotic lesions and lower resistance potential make this a better compound for a disease state modeled in C3HeB/FeJ mice and seen in human TB patients.

## Supplementary Material

Supplemental file 1
