# Supplementary material for: Efficacy and Improved Resistance Potential of a Cofactor-Independent InhA Inhibitor of Mycobacterium tuberculosis in the C3HeB/FeJ Mouse Model
Source: Antimicrob Agents Chemother. 2019 Mar 27;63(4):e02071-18. doi: 10.1128/AAC.02071-18 (PMC6496157; doi:10.1128/AAC.02071-18)
Supplement: Supplemental file 1 [file AAC.02071-18-s0001.pdf]

Supplemental Materials and Methods

Bacterial strains. *M. tuberculosis* Erdman (TMCC 107) was used for drug evaluations in mice. Bacteria were prepared for infection as described previously [1, 2].

Drug compounds. Isoniazid was purchased from Sigma. AN12855 was synthesized as described [3].

***In vivo* efficacy and resistance studies.** Female specific pathogen-free C3HeB/FeJ mice aged 8-10 weeks were purchased from Jackson Laboratories (Bar Harbor, ME). Mice were housed in a bio-safety level III animal facility and provided sterile bedding, water and mouse chow.

Ethics statement. The animal protocols involving mice were approved by Colorado State University's Institutional Animal Care and Use Committee.

Infection of mice. The *M. tuberculosis* Erdman strain (TMCC 107) was used for aerosol infections of mice, and the inocula were prepared as previously described [1]. C3HeB/FeJ mice were exposed to a low dose aerosol infection using a Glas-Col inhalation exposure system [2], resulting in an average of ~ 100 bacteria in the lungs of aerosolized mice 1 day following exposure.

Drug treatments of mice. For drug treatments, mice were administered isoniazid at 25 mg/kg in sterile water or AN12855 at 100 mg/kg in 1% [w/v] methyl cellulose (Sigma) 0.1% [v:v] polysorbate-80 (Sigma) via oral gavage five times weekly for 2, 4, or 8 weeks starting 68 days (efficacy studies) or for 7 consecutive days (PK studies).

Agar MICs

Agar MICs were determined using the agar proportion method using Middlebrook 7H11 with 0.02% [v/v] glycerol (Sigma), 10% [v/v] OADC (oleic acid, albumin, dextrose, catalase; Difco), cycloheximide (0.03 mg/mL), carbenicillin (0.05 mg/mL) [7H11-OADC] and further supplemented with two-fold increasing concentrations of each antimicrobial agent. The inoculum was $1x10^4$ CFU per 0.1 mL spot. MICs were scored as the first consecutive plate for which no growth was visible after at least 21 days of incubation at 37°C.

Enumeration of CFU burdens and drug-resistant colonies from infected lung tissues. Eight mice per group were used for studies involving C3HeB/FeJ mice owing to larger heterogeneity in treatment response. Infected mice were sacrificed at the start of treatment as pretreatment controls and to determine the *in vivo* frequency of resistance prior to the start of treatment. For endpoint analysis, mice were euthanized three days following the end of treatment, and the lungs were collected and frozen at -80°C. Whole lungs (C3HeB/FeJ mice) were disrupted with a tissue homogenizer (Bertin, Precellys), serially diluted in PBS, and plated on 7H11-OADC to determine lung CFU burdens. Samples were also replica plated onto a 7H11-OADC containing either isoniazid or AN12855 at 5× to 10× their respective agar MIC or a final concentration of 0.625 mg/L INH or 1.25 mg/L AN12855 to isolate *M. tuberculosis* Erdman resistant mutants. Colonies were counted after at least 21 days of incubation at 37°C. The viable bacterial counts of whole organs were calculated and converted to logarithms. The data were expressed as mean log10 CFU ± the standard error of the mean (SEM) for each group. In cases where no CFU were recovered, values were listed at the lower limit of detection. Frequency of resistance was calculated as the

number of resistant colonies over the total number of CFU plated. Companion studies evaluated the *in vitro* frequency of resistance using the same agar medium by plating serial dilutions of *M. tuberculosis* Erdman cultured to 2×10^9 CFU/mL in 7H9-ADC with 0.05% [v/v] Tween 80 (Sigma).

Isolation of DNA and sequencing of candidate genes. DNA was extracted from strains of interest using a Fastprep-24 (MP Biomedicals). Briefly, 1 ml of culture was transferred to a 2 ml Fastprep Lysing Matrix B tube and agitated for 20 s at a speed of 4 m/s. Extracts were centrifuged for 5 mins and then filtered through a 0.2 µM filter. Extracted DNA was stored at 4°C. PCR of candidate genes were performed using the following primer pairs fabG1-inhA (TB-FabG1-MMF1, TCAATACACCCGCAGCCA; InhA_F2, TTCATAGGTTCGGTCTCC; InhA-R1, GTGATACCCCACCGAAATGC) and katG (TB-KatG-MMF3, TCTATACCGGACTACGCC; TB-KatG-MMR4, TGGACCGTTTCGACAA).

Determination of compound MIC against drug resistant *M. tuberculosis*. The IC_{90} of compound was determined as previously described [4]. Briefly, bacterial growth was measured in the presence of test compounds. Compounds were prepared as 10-point two-fold serial dilutions in DMSO and diluted into 7H9 broth with 10% [v/v] OADC and 0.05% [w/v] tween 80 in 96-well plates with a final DMSO concentration of 2%. Each plate included assay controls for background (medium/DMSO only, no bacterial cells), zero growth (2 µM rifampicin) and maximum growth (DMSO only), as well as a rifampicin dose response curve. Growth was measured after 5 days by OD_{590}. Dose response curves were generated using the Levenberg-Marquardt algorithm and the concentrations that resulted in 90% inhibition of growth were determined (IC_{90}).

Lesion PK and bioanalysis. Drug levels in plasma, lung tissues and pulmonary lesions in drug treated C3HeB/FeJ mice were determined by bioanalytical analysis (PHARMout Laboratories, Inc.). Briefly, C3HeB/FeJ mice were infected by low dose aerosol and treatments started 10 weeks later. Mice received AN12855 (100 mg/kg) or INH (25 mg/kg) for seven consecutive days by oral gavage to achieve steady state levels in plasma and tissues. Plasma, whole lungs, fibrotic caseous lesions (Type I lesion), and excised caseum were harvested at defined intervals, weighed, and treated with methanol and acetonitrile at a ratio of 0.01 mL:0.44 mL per 0.05 mL of tissue homogenate to ensure sterility. Samples were kept frozen at -80°C until use. Antibiotic concentrations in extracted samples were analyzed by liquid chromatography-tandem mass spectrometry (LC-MS/MS) using an API5000 or 4000 instrument (AB Sciex). Quantification was achieved by comparing the analyte/internal standard peak areas with the internal standard. The limit of quantitation was 1 ng/mL.

1. Lenaerts, A.J., et al., Preclinical testing of the nitroimidazopyran PA-824 for activity against *Mycobacterium tuberculosis* in a series of in vitro and in vivo models. Antimicrob Agents Chemother, 2005. **49**(6): p. 2294-301.
2. Kelly, B.P., et al., Low-dose aerosol infection model for testing drugs for efficacy against *Mycobacterium tuberculosis*. Antimicrob Agents Chemother, 1996. **40**(12): p. 2809-12.
3. Xia, Y., et al., Discovery of a cofactor-independent inhibitor of *Mycobacterium tuberculosis* InhA. Life Science Alliance, 2018. **1**(3).
4. Ollinger, J., et al., A dual read-out assay to evaluate the potency of compounds active against *Mycobacterium tuberculosis*. PLoS One, 2013. **8**(4): p. e60531.
